# Ethnic disparities in acquiring 2009 pandemic H1N1 influenza: a case–control study

**DOI:** 10.1186/1471-2458-14-214

**Published:** 2014-03-01

**Authors:** Debeka Navaranjan, Laura C Rosella, Jeffrey C Kwong, Michael Campitelli, Natasha Crowcroft

**Affiliations:** 1 Department of Public Health Sciences, Public Health Ontario, 480 University Avenue, Suite 300, Toronto, Ontario M5G 1 V2, Canada; 2 Dalla Lana School of Public Health, University of Toronto, 155 College Street, Toronto, Ontario M5T 3 M7, Canada; 3 Institute for Clinical Evaluate Sciences, 2075 Bayview Avenue, Toronto, Ontario M4N 3 M5, Canada; 4 Department of Family and Community Medicine, University of Toronto, 500 University Avenue, Toronto, Ontario M5G 1 V7, Canada

**Keywords:** Influenza, Ethnicity, Epidemiology

## Abstract

**Background:**

Novel risk factors were associated with the 2009 pandemic A/H1N1 virus (pH1N1). Ethnicity was among these risk factors. Ethnic disparities in hospitalization and death due to pH1N1 were noted. The purpose of this study is to determine whether there are ethnic disparities in acquiring the 2009 pandemic H1N1.

**Methods:**

We conducted a test-negative case–control study of the risk of pH1N1 infection using data from Ontario, Canada. Cases were laboratory confirmed to have influenza using reverse-transcriptase polymerase chain reaction (RT-PCR), and controls were obtained from the same population and were RT-PCR negative. Multivariate logistic regression was used to determine the association between ethnicity and pH1N1 infection, while adjusting for demographic, clinical and ecological covariates.

**Results:**

Adult cases were more likely than controls to be self-classified as East/Southeast Asian (OR = 2.59, 95% CI 1.02-6.57), South Asian (OR = 6.22, 95% CI 2.01-19.24) and Black (OR = 9.72, 95% CI 2.29-41.27). Pediatric cases were more likely to be self-identified as Black (OR = 6.43, 95% CI 1.83-22.59). However, pediatric cases without risk factors for severe influenza infection were more likely to be South Asian (OR 2.92, 95% CI 1.11-7.68), Black (OR 16.02, 95% CI 2.85-89.92), and West Asian/Arab, Latin American or Multi-racial groups (OR 3.09 95% CI 1.06-9.00).

**Conclusions:**

pH1N1 cases were more likely to come from certain ethnic groups compared to test-negative controls. Insights into whether these disparities arise due to social or biological factors are needed in order to understand what approaches can be taken to reduce the burden of a future influenza pandemic.

## Background

Canada’s proportion of visible minorities has been increasing [1]. In 2006, 16.2% of Canada’s population was comprised of visible minorities, a rise from 13.4% in 2001 and 11.2% in 1996. Ontario has the largest proportion of visible ethnic minorities in Canada. Given the rise in visible minorities, it is imperative to consider ethnic differences in health in order to address potential disparities. In Canada, ethnic disparities have been identified for chronic diseases such as cardiovascular disease, diabetes, and obesity [2-4]. However, the relationship with infectious diseases, including influenza, is less clear.

There have been socio-economic disparities described for influenza. A study from the United States found that pediatric influenza-associated hospitalization was three times higher among high-poverty and high-crowding census tracts than in low-poverty and low-crowding census tracts [5]. Following the 2009 H1N1 pandemic, disparities associated with acquisition and severity of the 2009 pandemic A/H1N1 virus (pH1N1) were explored. A Canadian study exploring the relationship between social determinants of health and severity of pH1N1 found that hospitalization due to pH1N1 illness was associated with low education and living in neighbourhoods with high total or material deprivation [6]. Similarly, a study from England found that individuals from the most deprived quintile of the population had mortality rates due to pH1N1 three times greater than individuals from the least deprived quintile [7]. Studies from the United States have demonstrated ethnic disparities in incidence of influenza-like illness (ILI) during the 2009 H1N1 pandemic [8] and risk of hospitalization due to pH1N1 [9-11]. In the Canadian context, the frequency and severity of illness from pH1N1 were higher among First Nations individuals compared to the rest of the population [12-14]. However, few studies in Canada have examined the risk of acquiring pH1N1 among other ethnic minorities, where the distribution of ethnic minorities in Canada is different compared to other countries.

Clinically, groups at risk for infections, complications, hospitalization, and/or death due to influenza have been identified as children, elderly, and individuals with certain chronic medical conditions [15]. Social factors could also play a role in shaping risk groups for influenza. For example, ease of access to medical care, ability to limit risk of acquiring illness through social distancing practices, and acceptance of protective practices such as immunization can shape the groups at greater risk of acquiring influenza [16,17]. Currently, no ethnic groups are recognized as being at high risk for infections or subsequent complications due to seasonal influenza.

In this study we used a laboratory confirmed test-negative case–control study conducted during the pandemic to investigate the association between ethnicity and risk of acquiring pandemic H1N1.

## Methods

### Study design and population

We conducted a test-negative case–control study, which bases case status on laboratory confirmation of pH1N1 and controls on the same test being negative [18]. Our population was a clinic-based sample, consisting of individuals presenting to clinics for medical care. We collected information on symptoms at the time of testing to restrict our sample to those presenting with influenza-like illness, defined as an acute onset of respiratory illness with fever, cough and with one or more of the following: sore throat, arthralgia, myalgia, or prostration which is likely due to influenza. Ontario residents of all ages who had nasopharyngeal swabs for pH1N1 by real-time reverse-transcriptase polymerase chain reaction (RT-PCR) were eligible for inclusion. We identified study participants as having confirmed pH1N1 through testing at the Public Health Ontario (PHO) Laboratory or Mount Sinai Hospital/University Health Network. The PHO laboratory conducted 100% of the testing in the first months of the pandemic. Later on, a few other laboratories in major centres, the largest being Mount Sinai Hospital/University Health Network, conducted testing as well.

We included individuals with a test date between April 13, 2009 and July 20, 2009 (pandemic wave 1). The study was approved by the Research Ethics Board of University of Toronto, Toronto, Ontario, Canada.

### Study variables

Trained interviewers from the Institute for Social Research at York University, Toronto conducted standardized telephone interviews between August and September 2009 to obtain information on demographics, health status, symptoms, and treatment, including hospitalization. For children 14 and under, interviews were completed by a parental proxy.

Individuals were asked to self-identify with an ethnicity/ethnicities. Ethnicity was based on the question, “People in Canada come from many different ethnic and racial backgrounds. I would like you to identify which ethnicity best describes you. You may choose more than one category.” Ethnicities were then read aloud to the participants. The ethnic groupings were based on the categories similar to Statistics Canada definitions: White, East/Southeast Asian, South Asian, Black, West Asian/Arab, Latin American, and Other. Individuals who identified with multiple ethnic groups were classified as Multi-Racial person. West Asian/Arab, Latin American and Multi-racial were combined with Other because of small sample sizes for these ethnic groups. Aboriginals could not be included in this analysis because of lack of data (<1% of the sample).

Conceptual models of the association between ethnicity and acquisition of pH1N1 were developed *a priori* to guide the model building. This enabled the consideration of potential confounders and effect modifiers. The variables considered for adults were age, gender, current smoking status, number of visits to their regular primary care doctor in the prior 12 months (low, ≤1; moderate, 2–6; high, >6), completion of post-secondary education, immigrant status (non-immigrant, 0–5 years in Canada, >5 years in Canada), whether there were children in the household, household density (the ratio of the number of individuals in a household to the number of sleeping rooms), previous seasonal influenza vaccination, residence in Toronto (the capital and largest metropolitan city in Ontario), healthcare worker status, and testing date before or after June 11th, 2009 (the date when clinicians in Ontario were instructed to switch from submitting samples from all patients presenting with influenza-like illness to only submitting samples from patients at high risk of complications or for clinical management of hospitalized cases) [19]. In addition to the individual level variables collected through the survey, we examined the impact of deprivation captured through the Deprivation Index (DI) developed by Institut national de santé publique du Québec (INSPQ) [6,20,21]. The DI includes social deprivation, material deprivation, total deprivation, employment rate, proportion of the population with less than high school education, and prevalence with low income, where individuals were classified as living in an area with a high prevalence of low income if they resided in dissemination areas that were in the top quintile for low income of the population study. We also considered if individuals were tested in Toronto since this represents the major urban centre of our study population as well as contains the highest proportion of visible minorities and other ethnic groups. Underlying medical conditions were analyzed as a binary variable (≥1 underlying medical conditions, which included diabetes, cancer, heart disease, asthma, chronic obstructive pulmonary disease (COPD), immune disorder, liver disease, kidney or renal disease, splenectomy, sickle cell anemia, seizure disorder, leukemia, cystic fibrosis, cerebral palsy), and obesity was considered separately. In children, all the same variables were considered, with underlying medical conditions restricted to those commonly observed in children (≥ 1 of either asthma or immune disorder).

### Statistical analysis

We first tested the interaction between ethnicity and being a child (< 18 years of age) in order to determine if appropriate to pool adults and children. Given the significant interaction and the different confounders, we then had to stratify all analysis according <18 years of age versus ≥ 18 years of age. Descriptive statistics (means and proportions) were calculated for all variables. In order to develop the multivariable model, the Hosmer and Lemeshow forward model building strategy was used [22]. Briefly, odds ratios were obtained from univariate logistic regression to identify risk factors for acquisition of pH1N1. Variables were then entered into the model based on their effect size and p-value. Variables that had p-values <0.05 were included in the final model, as well as variables that had a significant (>10%) effect on any of the ethnic group’s OR. Age and gender were forced into the multivariate model, as these were deemed important variables to consider for the association. Gender was *a priori* identified as an important effect modifier and therefore the interaction was tested. At the end of this iterative model building process, a verification step was performed by adding any variable not selected for the original multivariate model one at a time to identify variables that, by themselves, were not influential but may have made an important contribution in the presence of other variables.

In addition, the following sensitivity analyses were performed: including individuals who were tested because of recent travel to Mexico; including individuals who did not present with symptoms at testing; no restrictions implemented (no exclusions); stratifying by presence of risk factors; stratifying by testing prior to June 11th, 2009; and according to Toronto residence.

## Results

A total of 402 adults and 352 children were included. There were 229 controls and 173 cases among adults, and 112 controls and 240 cases among children. The demographic, clinical, and ecologic-level characteristics are described in Table 1. Among cases, more than half were females and 59% of adult cases and 40% of pediatric cases were of white ethnicity.

**Table 1:** Characteristics of the adult and pediatric study population by case status

			**Adults**				**Children**					
		**Total study population**		**Tested negative**		**Tested positive**		**Total study population**		**Tested negative**		**Tested positive**
		**(N = 402)**		**(N = 229)**		**(N = 173)**		**(N = 352)**		**(N = 112)**		**(N = 240)**
**Variable**	**N**	**%**	**N**	**%**	**N**	**%**	**N**	**%**	**N**	**%**	**N**	**%**
Ethnicity*												
White	266	66.2	164	71.6	102	59.0	166	47.2	70	62.5	96	40.0
East/Southeast Asian	42	10.5	23	10.0	19	11.0	47	13.4	11	9.8	36	15.0
South Asian	32	8.0	11	4.8	21	12.1	47	13.4	14	12.5	33	13.8
Black	19	4.7	5	2.2	14	8.1	35	9.9	5	4.5	30	12.5
Other	35	8.7	20	8.7	15	8.7	49	13.9	10	8.9	39	16.3
**Age**												
<5	N/A		N/A		N/A		94	26.7	45	40.2	49	20.4
5-11	N/A		N/A		N/A		135	38.4	31	27.7	104	43.3
12-17	N/A		N/A		N/A		123	34.9	36	32.1	87	36.3
18-34	150	37.3	79	34.5	71	41.0	N/A		N/A		N/A	
35-49	124	30.9	71	31.0	53	30.6	N/A		N/A		N/A	
50-64	95	23.6	58	25.3	37	21.4	N/A		N/A		N/A	
65+	33	8.2	21	9.2	12	6.9	N/A		N/A		N/A	
**Gender (female)**	241	60.0	139	60.7	102	59.0	246	69.9	80	71.4	166	69.2
**BMI class**												
Normal weight (18.5 ≤ BMI <25)	187	46.5	111	48.5	76	43.9	115	32.7	38	33.9	77	32.1
Overweight (25 ≤ BMI < 30)	114	28.4	62	27.1	52	30.1	32	9.1	8	7.1	24	10.0
Obese (BMI ≥ 30)	72	17.9	38	16.6	34	19.7						
**Current smoker**	110	27.4	70	30.6	40	23.1	4	1.14	2	1.79	2	0.83
Missing	N/A		N/A		N/A		214	60.8	74	66.07	410	58.3
**Number of family doctor visits in the past year**												
<1	73	18.2	45	19.7	28	16.2	78	22.2	20	17.9	58	24.2
2 to 6	257	63.9	152	66.4	105	60.7	200	56.8	68	60.7	132	55.0
>6	72	17.9	32	14.0	40	23.1	74	21.0	24	21.4	50	20.8
**Underlying medical conditions**^**†**^	86	21.4	42	18.3	44	25.4	56	15.9	20	17.9	36	15.0
**Material deprivation**^**‡**^												
1 (low)	95	23.6	63	27.5	32	18.5	71	20.2	26	23.2	45	18.8
2	87	21.6	46	20.1	41	23.7	74	21.0	26	23.2	48	20.0
3	65	16.2	37	16.2	28	16.2	67	19.0	23	20.5	44	18.3
4	58	14.4	28	12.2	30	17.3	70	19.9	19	17.0	51	21.3
5 (high)	73	18.2	45	19.7	28	16.2	56	15.9	18	16.1	38	15.8
**Social deprivation**^**‡**^												
1 (low)	84	20.9	47	20.5	37	21.4	105	29.4	37	33.0	68	28.3
2	69	17.2	35	15.3	34	19.7	73	20.7	23	20.5	50	20.8
3	74	18.4	44	19.2	30	17.3	60	17.1	20	17.9	40	16.7
4	84	20.9	51	22.3	33	19.1	58	16.5	19	17.0	39	16.3
5 (high)	67	16.7	42	18.3	25	14.5	42	11.9	13	11.6	29	12.1
**Total deprivation category**^**‡**^												
1 (low)	71	17.7	38	16.6	33	19.1	77	21.9	30	26.8	47	19.6
2	257	63.9	153	66.8	104	60.1	219	62.2	72	64.3	147	61.3
3 (high)	50	12.4	28	12.2	22	12.7	42	11.9	10	8.9	32	13.3
**Individuals living in a neighbourhood with low employment rate**^**§**^	88	21.9	53	23.1	35	20.2	57	16.2	15	13.4	42	17.5
**Individuals residing in a neighbourhood with high proportion of low income**^**§**^	100	24.9	56	24.5	44	25.4	74	21.0	13	11.6	61	25.4
**High school or less education**	58	14.4	35	15.3	23	13.3	53	15.1	16	14.3	37	15.4
**Post-secondary education completed**	261	64.9	153	66.8	108	62.4	250	71.0	77	68.8	173	72.1
**Household density (median)**^ǁ^		1.0		1.0		1.0		1.3		1.2		1.3
**Children in household**												
0	231	57.5	144	62.9	87	50.3	N/A		N/A		N/A	
1	170	42.3	84	36.7	86	49.7	247	70.2	79	70.5	168	70.0
2	N/A		N/A		N/A		79	22.4	25	22.3	54	22.5
3	N/A		N/A		N/A		24	6.8	7	6.3	17	7.1
**Receipt of 2008 seasonal vaccine**	156	38.8	83	36.2	73	42.2	108	30.7	25	22.3	83	34.6
**Tested before June 11th, 2009**	315	78.4	17	7.4	103	59.5	251	71.3	112	100.0	139	57.9
**Healthcare provider**^**¶**^	48	11.9	23	10.0	25	14.5	N/A		N/A		N/A	
**Resides in Toronto**	121	30.1	84	36.7	37	21.4	78	22.2	24	21.4	54	22.5
**Immigrant category**												
Non-Immigrant	251	62.4	148	64.6	103	59.5	316	89.8	106	94.6	210	87.5
Immigrant	N/A		N/A		N/A		36	10.2	6	5.4	30	12.5
Immigrant, settled for 0–5 years	24	6.0	9	3.9	15	8.7	N/A		N/A		N/A	
Immigrant, settled for >5	121	30.1	67	29.3	54	31.2	N/A		N/A		N/A	
**Hospitalized**	64	15.9	0	0	64	37.0	79	22.4	0	0	79	32.9

*This was based on the question “People in Canada come from many different ethnic and racial backgrounds. I would like you to identify which ethnicity best describes you. You may choose more than one category.” The ethnic groupings were based on the categories similar to Statistics Canada definitions.

^†^Underlying medical conditions for adults was having at least one of the following: diabetes, cancer, heart disease, asthma, chronic obstructive pulmonary disease (COPD), immune disorder, liver disease, kidney or renal disease, splenectomy, sickle cell anemia, seizure disorder, leukemia, cystic fibrosis, or cerebral palsy. In children, underlying medical conditions was having either asthma or immune disorder.

^‡^Material Deprivation was based on employment to population ratio, average income and proportion without high school diploma. Social deprivation was based on proportion of persons living alone; proportion separated, divorced or widowed; and proportion of single-parent families. Quintiles of material and social deprivation were created based on dissemination areas in Ontario, with a score of 1 representing low deprivation and 5 indicating high deprivation. The material and social deprivation were combined to form a total deprivation score, which was categorized as high, middle or low deprivation.

^§^Low employment rate and High Proportion of Low Income are based on individuals residing in a dissemination area in Ontario. Individuals living in a neighbourhood in the bottom quintile of all dissemination areas for low employment/with high proportion of low income, based on our study population, were classified as being in the low employment rate/high proportion of low income categories.

^ǁ^Calculated as the number of people in the household divided by the number of sleeping rooms in the household.

^¶^Healthcare provider is defined as a doctor, nurse or another healthcare professional.

N/A = Not applicable.

In adults, cases were more likely than controls to be South Asian (OR 3.07, 95% CI 1.42-6.63) or Black (OR 4.50, 95% CI 1.57-12.87), have greater than moderate material deprivation (4th quintile) (OR 2.11, 95% CI 1.08-4.11), have children in the household (OR 1.70, 95% CI 1.13-2.53), or be a recent immigrant (2.40, 95% CI 1.01-5.68) (Table 2).

**Table 2:** Unadjusted odds ratios for pH1N1 influenza acquisition for various potential risk factors in adults and children

		**Adults**		**Children**
**Variable**		**OR (95% CI)**		**OR (95% CI)**
Ethnicity*	White	ref		ref
	East/Southeast Asian	1.33 (0.69-2.56)		2.39 (1.14-5.01)
	South Asian	3.07 (1.42-6.63)		1.72 (0.86-3.45)
	Black	4.50 (1.57-12.87)		4.38 (1.62-11.84)
	Other	1.21 (0.59-2.46)		2.84 (1.33-6.08)
**Age**	18-34	ref	<5	0.45 (0.26-0.79)
	35-49	0.83 (0.52-1.34)	5-11	1.39 (0.79-2.43)
	50-64	0.71 (0.42-1.20)	12-17	ref
	65+	0.64 (0.29-1.39)		
**Gender (Female)**		0.93 (0.62-1.39)		0.90 (0.55-1.47)
**BMI class**	II (18.5 ≤ BMI <25)	ref		ref
	III (25 ≤ BMI < 30)	1.23 (0.77-1.96)		1.48 (0.61-3.60)
	IV (BMI ≥ 30)	1.31 (0.76-2.26)		
**Current smoker**		0.68 (0.43-1.07)		0.96 (0.91-1.02)
**Number of visits to family doctor in the past year**	Low (<=1)	0.90 (0.53-1.54)		1.49 (0.83-2.69)
	Moderate (2–6)	ref		ref
	High (>6)	1.81 (1.07-3.07)		1.07 (0.61-1.90)
**Underlying medical conditions**^**†**^		1.51 (0.93-2.44)		0.81 (0.44-1.48)
**Material deprivation**^**‡**^	1 (low)	ref		ref
	2	1.76 (0.96-3.19)		1.07 (0.54-2.10)
	3	1.49 (0.78-2.85)		1.11 (0.55-2.22)
	4	2.11 (1.08-4.11)		1.55 (0.76-3.17)
	5 (high)	1.23 (0.65-2.31)		1.22 (0.58-2.56)
**Social deprivation**^**‡**^	1 (low)	ref		ref
	2	1.23 (0.65-2.34)		1.18 (0.63-2.23)
	3	0.87 (0.46-1.63)		1.09 (0.56-2.13)
	4	0.82 (0.45-1.52)		1.12 (0.57-2.20)
	5 (high)	0.76 (0.39-1.46)		1.21 (0.56-2.61)
**Total deprivation category**^**‡**^	1 (low)	ref		ref
	2	0.78 (0.46-1.33)		1.30 (0.76-2.23)
	3 (high)	0.91 (0.44-1.87)		2.04 (0.88-4.75)
**Individuals living in a neighbourhood with low employment rate**^**§**^		0.84 (0.52-1.36)		1.37 (0.73-2.60)
**Individuals residing in a neighbourhood with a high proportion of low income**^**§**^		1.05 (0.67-1.66)		2.60 (1.36-4.96)
**High school or less education**		0.85 (0.48-1.50)		1.09 (0.58-2.06)
**Post-secondary school completion**		0.82 (0.54-1.24)		1.21 (0.74-1.98)
**Household density**^**ǁ**^		1.26 (0.84-1.87)		1.81 (0.95-3.44)
**Children in household**		1.70 (1.13-2.53)	1	ref
			2	1.02 (0.59-1.75)
			>3	1.14 (0.46-2.87)
**Receipt of 2008 seasonal vaccine**		1.25 (0.84-1.88)		1.96 (1.16-3.31)
**Tested prior to June 11th, 2009**		0.12 (0.07-0.21)		N/A
**Healthcare provider**^**¶**^		1.51 (0.83-2.77)		N/A
**Toronto**		0.47 (0.30-0.74)		1.07 (0.62-1.83)
**Immigrant category**				
Non-Immigrant		ref		ref
Immigrant		N/A		2.52 (1.02-6.25)
Immigrant, Settled for 0–5 years		2.40 (1.01-5.68)		N/A
Immigrant, Settled for >5		1.16 (0.75-1.80)		N/A

*This was based on the question “People in Canada come from many different ethnic and racial backgrounds. I would like you to identify which ethnicity best describes you. You may choose more than one category.” The ethnic groupings were based on the categories similar to Statistics Canada definitions.

^†^Underlying medical conditions for adults was having at least one of the following: diabetes, cancer, heart disease, asthma, chronic obstructive pulmonary disease (COPD), immune disorder, liver disease, kidney or renal disease, splenectomy, sickle cell anemia, seizure disorder, leukemia, cystic fibrosis, or cerebral palsy. In children, underlying medical conditions was having either asthma or immune disorder.

^‡^Material Deprivation was based on employment to population ratio, average income and proportion without high school diploma. Social deprivation was based on proportion of persons living alone; proportion separated, divorced or widowed; and proportion of single-parent families. Quintiles of material and social deprivation were created based on dissemination areas in Ontario, with a score of 1 representing low deprivation and 5 indicating high deprivation. The material and social deprivation were combined to form a total deprivation score, which was categorized as high, middle or low deprivation.

^§^Low employment rate and High Proportion of Low Income are based on individuals residing in a dissemination area in Ontario. Individuals living in a neighbourhood in the bottom quintile of all dissemination areas for low employment/with high proportion of low income, based on our study population, were classified as being in the low employment rate/high proportion of low income categories.

^ǁ^Calculated as the number of people in the household divided by the number of sleeping rooms in the household.

^¶^Healthcare provider is defined as a doctor, nurse or another healthcare professional.

N/A = Not applicable.

Among children, cases were more likely than controls to be East/Southeast Asian (OR 2.39, 95% CI 1.14-5.01), Black (OR 4.38, 95% CI 1.62-11.84) and either Latin American, West Asian/Arab or Multi-racial ethnicity (OR 2.84, 95% CI 1.33-6.08), be from a neighbourhood with a high proportion of individuals with low income (OR 2.60, 95% 1.36-4.96), have obtained the 2008 seasonal vaccine (OR 1.96, 95% 1.16-3.31), or be an immigrant (OR 2.52, 95% CI 1.02-6.25).

In multivariate analysis, adjusting for all significant confounders identified through our model building strategy increased the observed ORs for all ethnic groups (Table 3). After adjustment, for adults, cases were more likely to be East/Southeast Asian (OR 2.59, 95% CI 1.02-6.57), South Asian (OR 6.22, 95% CI 2.01-19.24), and Black (OR 9.72, 95% CI 2.29-41.27). The sensitivity analyses indicate that including individuals who had travelled to Mexico increased the estimates for all ethnic groups. In addition, the analysis was conducted without any restrictions and this resulted in a slight increase in ORs for East/Southeast Asian (OR 2.96, 95% CI 1.24-7.05), South Asian (OR 7.42, 95% CI 2.79-19.74), and Black (OR 11.28, 95% CI 2.85-44.60). Among those who did not have risk factors, the point estimates for the odds ratio after adjustment increased for South Asians; the confidence intervals for these estimates were wider given the reduced samples size after stratifying. As noted, the risk of diabetes and heart disease differs by ethnicity, hence, these medical conditions were considered separately as a group in the same analysis, and they did not appreciably change the effect sizes.

**Table 3:** Odds ratios from multivariate logistic regression models for pH1N1 illness acquisition and ethnicity under various sensitivity analyses

	**Adults**	**Children**
Ethnic group*	**Adjusted OR (95% CI)**^**†**^	**Adjusted OR (95% CI)**^**‡**^
**Restricted to those with symptoms and those tested for reasons other than travel to Mexico (primary analysis)**		
White	ref	ref
East/Southeast Asian	2.59 (1.02-6.57)	1.96 (0.82-4.66)
South Asian	6.22 (2.01-19.24)	1.88 (0.80-4.41)
Black	9.72 (2.29-41.27)	6.44 (1.83-22.59)
Other	2.00 (0.74-5.42)	2.06 (0.83-5.11)
**Individuals tested because of travel to Mexico added**		
White	ref	ref
East/Southeast Asian	2.99 (1.20-7.45)	2.01 (0.85-4.73)
South Asian	7.34 (2.42-22.28)	1.84 (0.80-4.24)
Black	10.93 (2.61-45.73)	6.45 (1.86-22.44)
Other	2.24 (0.88-5.70)	1.70 (0.73-3.97)
**Those not presenting with symptoms are included**		
White	ref	ref
East/Southeast Asian	2.57 (1.06-6.23)	1.92 (0.84-4.38)
South Asian	6.03 (2.21-16.44)	1.83 (0.81-4.14)
Black	10.34 (2.58-41.46)	5.44 (1.72-17.24)
Other	1.67 (0.70-3.99)	2.28 (0.95-5.45)
**No restrictions**		
White	ref	ref
East/Southeast Asian	2.96 (1.24-7.05)	1.99 (0.88-4.50)
South Asian	7.42 (2.79-19.74)	1.82 (0.82-4.06)
Black	11.28 (2.85-44.60)	5.62 (1.78-17.71)
Other	1.79 (0.78-4.08)	1.80 (0.81-4.01)
**Heart disease and diabetes added independently of other underlying medical conditions**		
White	ref	
East/Southeast Asian	2.56 (0.99-6.64)	
South Asian	6.55 (1.99-21.57)	
Black	10.19 (2.35-44.22)	
Other	1.34 (0.46-3.90)	
**Without risk factors**		
White	ref	ref
East/Southeast Asian	2.63 (0.91-7.59)	2.48 (0.92-6.64)
South Asian	10.34 (2.39-44.80)	2.92 (1.11-7.68)
Black	11.90 (2.30-61.65)	16.02 (2.85-89.92)
Other	1.69 (0.54-5.30)	3.09 (1.06-9.00)
**Tested prior to June 11th, 2009**		
White	ref	ref
East/Southeast Asian	2.66 (1.01-7.02)	2.90 (1.10-7.64)
South Asian	6.97 (2.07-23.50)	1.82 (0.68-4.87)
Black	8.18 (1.85-36.22)	7.21 (1.72-30.21)
Other	2.05 (0.70-6.05)	2.34 (0.82-6.71)

*This was based on the question “People in Canada come from many different ethnic and racial backgrounds. I would like you to identify which ethnicity best describes you. You may choose more than one category.” The ethnic groupings were based on the categories similar to Statistics Canada definitions.

^†^For adults, all models were adjusted for age, sex, children in household, material deprivation, chronic conditions, receipt of 2008 seasonal vaccine, tested prior to June 11th, 2009, residing in Toronto, length of stay in Canada.

^‡^For children, all models were adjusted for age, sex, total deprivation, receipt of 2008 seasonal vaccine, high proportion of neighbourhood with low income, material deprivation, post-secondary education, chronic condition, residence in Toronto.

In multivariate analysis for children, cases were more likely to be Black (OR 6.43, 95% CI 1.83-22.59) (Table 3). Adjusting for confounders increased the observed OR for Black ethnicity. When the analysis included individuals who did not have symptoms at time of testing, the estimates did not change for any ethnicity except for the Black group (OR 5.44, 95% CI 1.71-17.24). For the analysis conducted without any restrictions (no exclusions), the estimates did not change appreciably. When stratifying by presence of risk factors, among those without risk factors, the estimates increased for all ethnic groups, but the greatest change was observed for the Black group. Cases were more likely to be South Asian (OR 2.92, 95% CI 1.11-7.68), Black (OR 16.02, 95% CI 2.85-89.92), or West Asian/Arab, Latin American, or Multi-racial (OR 3.09 95% CI 1.06-9.00). The ORs for ethnic groups were higher for adults who resided outside Toronto compared to those who resided in Toronto (Table 4). This effect was seen for all ethnic groups, except East/Southeast Asian. This trend was also observed in children but both failed to reach statistical significance (results not shown). Testing date was controlled for in the multivariate model, however, since many of the controls were tested after June 11, we ran a sensitivity analyses restricting to only controls tested prior to June 11, and aside from increasing confidence intervals the parameter estimates for ethnicity were relatively unchanged.

**Table 4:** A multivariate logistic regression model for pH1N1 illness acquisition in adults and ethnicity, stratified by Toronto residence

	**Wave 1**
Ethnic group*	**OR (95% CI)**^**†**^
**Not Residing in Toronto**	
White	ref
East/Southeast Asian	1.86 (0.57-6.08)
South Asian	9.95 (2.17-45.59)
Black	14.61 (1.49-143.36)
Other	2.26 (0.68-7.53)
**Residing in Toronto**	
White	ref
East/Southeast Asian	6.80 (1.02-45.51)
South Asian	2.32 (0.29-18.79)
Black	4.20 (0.38-46.46)
Other	1.50 (0.17-13.48)

*This was based on the question “People in Canada come from many different ethnic and racial backgrounds. I would like you to identify which ethnicity best describes you. You may choose more than one category.” The ethnic groupings were based on the categories similar to Statistics Canada definitions.

^†^For adults, all models were adjusted for age, sex, children in household, material deprivation, chronic conditions, receipt of 2008 seasonal vaccine, tested prior to June 11th, 2009, length of stay in Canada

## Discussion

In this case–control study we found evidence of ethnic disparities in acquiring the 2009 pandemic H1N1 such that among adults lab-confirmed pH1N1 cases were more likely to be of East/Southeast Asian, South Asian and Black ethnicity compared to test-negative controls. This association persisted after controlling for several important confounders, including individual and ecological socioeconomic measures and clinical risk factors, and under a variety of restrictions imposed as part of a sensitivity analysis. Among children, not all ethnic disparities seen in adults were detected, however consistent with adults, pediatric cases were more likely to be classified as Black compared to other ethnic groups after controlling for confounders.

Previous studies have found ethnic discrepancies when looking at the distribution of pH1N1 illnesses. In Utah, U.S., it was reported that 29.5% of pH1N1 hospitalized cases were minorities (those not classified as non-Hispanic Whites), significantly greater than the mean of 22% of hospitalized cases that were minorities during the previous three influenza seasons [9]. The majority of studies have looked at hospitalization rates due to pH1N1 and ethnic disparities. For example, in Chicago, U.S., Asian/Pacific Islanders, the non-Hispanic Black group, and the Hispanic group had higher rates of hospitalization due to pH1N1 compared to the non-Hispanic White group [10]. Also, in Wisconsin, U.S., it was reported that the groups classified as Black, Hispanic, and Asian had several-fold higher hospitalization than the non-Hispanic White group due to pH1N1 [11]. Similar findings have also been reported in the United Kingdom [23]. Social factors may explain the ethnic disparities seen in this and other studies, which may operate through a variety of mechanisms. Social factors such as crowding, use of public transit, and ability to keep children away from other groups of children during school or day-care centre closures during a pandemic can play a role in increasing exposure risk [16]. A study in the U.S. compared social risk factors, including social distancing across ethnicities, and found that those identifying as Black and Hispanic (both Spanish and English speaking) found it more difficult to avoid public transportation than non-Hispanic White [17]. Also, those identifying as Black and Spanish-speaking Hispanic reported greater challenges to finding childcare that separated their children from other children, compared to those identifying as non-Hispanic White. There may also be differential access to information regarding the risks associated with influenza as well as preventive measures. For example, we have evidence from seasonal vaccination programs that certain ethnic groups are less likely to receive seasonal influenza vaccines [24,25].

This study does have a few limitations. First, we were somewhat limited in sample size for certain ethnic groups, resulting in wide confidence intervals observed for adults and children who identified their ethnicity as Black and South Asian. Including all variables in the multivariate model did slightly increase the width of the confidence intervals, however, the variables included were all important confounders and thus failing to control for these variables would produce confounded results. In addition, most of the variables are based on self-report and thus are susceptible to misclassification. Healthcare seeking behaviour was represented by number of primary care physician visits in the last 12 months. However, healthcare seeking behaviour could also be measured by the resources and services that are available to ethnic groups within their own communities. It is important to note that all individuals in this study sought care, thus minimizing the bias that access would have on the results. Another limitation is that a few of the variables were not available at the individual level, such as household income and employment, which are important confounders to consider for studying this relationship. Ecological-level variables were used to assess impact of income based on prevalence of low income among total persons in household within a dissemination area. It should also be noted that these findings were specific to our study, and they may be different for other studies with different ethnic compositions.

## Conclusions

The purpose of this study was to determine whether there are ethnic disparities in acquiring the 2009 pandemic H1N1, and this study did find evidence for such disparities. Identifying ethnic differences in infectious disease risk is critical to understanding how best to reduce any excess burden experienced by these populations. To minimize risk in these ethnic groups, further insight needs to be gained as to whether these findings are a result of social or biological factors, or both. The understanding of the origin of these disparities in acquiring pH1N1 can help us to promote and plan interventions accordingly to reduce the burden of illness among ethnic groups.

## Competing interests

The authors declare that they have no competing interests.

## Authors’ contributions

LR and NC were responsible for conception and design. DN and LR performed the statistical analyses. All of the authors contributed in the interpretation and the discussion of the results. DN wrote the first draft of the paper. All of the authors critically reviewed and contributed to the final draft. All authors read and approved the final manuscript.

## Pre-publication history

The pre-publication history for this paper can be accessed here:

http://www.biomedcentral.com/1471-2458/14/214/prepub
